# Evaluation of antibiotic-induced taste and smell disorders using the FDA adverse event reporting system database

**DOI:** 10.1038/s41598-021-88958-2

**Published:** 2021-05-05

**Authors:** Yusuke Kan, Junko Nagai, Yoshihiro Uesawa

**Affiliations:** 1 Department of Medical Molecular Informatics, Meiji Pharmaceutical University, Tokyo, 204-8588 Japan; 2 Nanohana Pharmacy, Tokyo, 196-0014 Japan

**Keywords:** Medical research, Epidemiology

## Abstract

Adverse effects can occur owing to anorexia, which can reduce treatment compliance and worsen the patients overall condition. One such side effect, namely drug-induced taste and smell disorders, reduces patients quality of life. Although antibiotics can cause taste and smell disorders, a few studies have examined antibiotic-induced taste and smell disorders. Therefore, this study comprehensively analyzed the relationship between taste and smell disorders and antibiotic usage. The side effects of antibiotics were investigated using the FDA Adverse Event Reporting System database (FAERS). The reporting odds ratios between the listed drugs and taste and smell disorders *P* values were comprehensively calculated. Adjusted odds ratios were calculated to account for patient background. Furthermore, to clarify the feature of this adverse effect, shape parameters indicating the expression pattern were calculated. Signals that induced taste and smell disorders were detected for six antibiotics, including drugs for which this event is not described in the package insert in Japan. Multiple logistic regression analysis suggested an association of taste and smell disorders with gender, hypertension, mental disorder, and cancer. The median time to onset of antibiotic-induced taste and smell disorders was 2–5 days. Six antibiotics could be analyzed, and four of these drugs matched those with detected signals. Our study supported previous findings on gender and age. Furthermore, antibiotic-induced taste and smell disorders are likely to develop in the early stage of treatment. For these reasons, it is important to remember the risk of developing of taste and smell disorders when administering antibiotics. In addition, it is recommended that the patient be monitored carefully for at least 1 week before initiating treatment, and the patients course should be followed for at least 2 months.

## Introduction

Although taste and smell disorders are not a life-threatening adverse event (AE), it is believed to reduce patients’ quality of life and decrease treatment compliance. Furthermore, in severe cases, anorexia can affect the patients general condition. In the American National Health and Nutrition Examination Survey 2011–2012 involving 140 million adult respondents, more than 5% experienced taste and smell disorders, and it has been reported that 10% of patients have experienced the olfactory disorder. Patients with smell disorders are often aware of taste disorders; however, several patients confuse taste disorder with smell disorder, which is a taste disorder. Furthermore, chronic kidney disease, heart disease, malignant tumors, psychiatric disorders, and depression are chronic diseases associated with taste and smell disorders^1–3^. To date, 50% of patients using levofloxacin inhalation therapy have been reported to possibly have taste and smell disorders. In another study, dysgeusia was reported in patients receiving a combination of ciprofloxacin and tinidazole^4^.

Drug-induced taste disorder in Japan accounts for about 16% of outpatient cases, and smell disorder account for approximately 3%. It has also been reported that standard zinc replacement therapy requires a longer period of treatment than other causes^5,6^. In Japan, the incidence of predominant dysgeusia peaked in patients in their 50 s and 60 s in the 1980s, but it has recently been reported that the onset is more frequent after the 60 s decade of life^7^.

Chemotherapeutic agents, other anticancer drugs, ACE inhibitors, and antibiotics have been reported to induce dysgeusia^6,7^. Whereas the drugs are used only for specific patient groups, antibiotics may be used in most patients. Analysis of the time to onset of taste and smell disorders during macrolide and fluoroquinolone therapy has shown that most taste and smell disorders developed within one month of taking the antibiotics. In addition, they report that in severe cases of taste and smell disorders caused by macrolides and fluoroquinolones, there can be a permanent taste and smell disorders. When attributed to macrolides, 58% took more than a week to disappear. However, it was reported that in 7% of cases, taste and smell disorders did not disappear after 150 days of taking the drug^8^. The study by Marco et al. (2011) provided an analysis of such AEs by class of antibiotic. However, no detailed analysis of each drug was reported. In this study, by clarifying the relationship between taste and smell disorders and each antibiotic, we aim to obtain information that contributes to reducing AEs. We used the FDA Adverse Event Reporting System (FAERS) to assess AEs based on the reporting odds ratio (ROR)^9–13^. We examined the relationship between antibiotics and taste and smell disorders by examining the number of reports. In addition, we examined the covariates such as patient background using adjusted odds ratios (AdORs) via multiple logistic regression analysis^14,15^. In addition, using time information data, an analysis of AE onset time using weibull distribution was performed to examine the AE onset time profile for each drug^16–18^. This is the first report to comprehensively analyze the relationship between antibiotics and taste and smell disorders.

## Results

**Figure 1:**
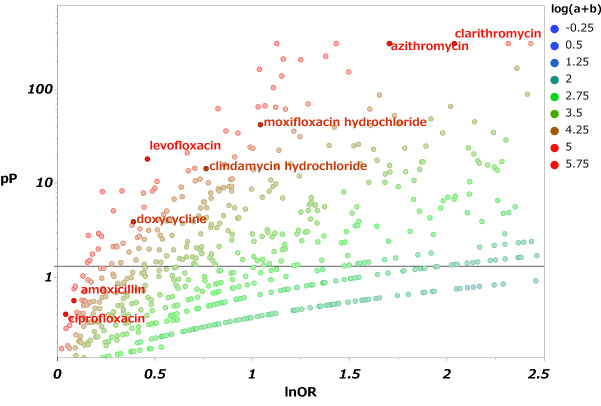
Taste and smell disorders associated with pharmaceuticals. This figure presents the relationships between taste and smell disorders and suspected causative drugs. The X-axis is the logarithm of the reporting odds ratio (lnOR), and the Y-axis is the negative logarithm of the *P* value calculated using Fisher's exact test (− log *P* value; pP). The horizontal line in the figure indicates the reference line at pP = 1.3 (*P* = 0.05). a + b indicates the number of reports of taste and smell disorders, and increasing feature is represented by a change in color from blue to red.

A scatter plot of the relationship between the ROR and statistically significant differences, a volcano plot, was made to examine antibiotics that caused taste and smell disorders (Fig. 1). In this figure, the drugs in the upper right of the scatter plot indicate more likely to cause taste and smell disorders.

The total number of reports in the FAERS database was 43,955,583, including 78,974 reports of taste and smell disorders. The lower limit of the 95% confidence interval (CI) of ROR for these antibiotics exceeded 1, indicating that a signal was detected (Table 1). Multiple components of these antibiotics have been reported (see Supplementary Table S1).

**Table 1:** ROR of antibiotics that cause taste and smell disorders.

Drug	ROR	95%CI	*P* value	a	b	c	d
Amoxicillin	1.09	(0.94–1.26)	0.2744	179	91,597	78,795	43,785,012
Azithromycin	5.5	(5.15–5.87)	< .0001	911	93,005	78,063	43,783,604
Ciprofloxacin	1.04	(0.95–1.15)	0.3962	397	211,546	78,577	43,665,063
Clarithromycin	7.67	(7.22–8.16)	< .0001	1045	76,575	77,929	43,800,034
Clindamycin	2.15	(1.81–2.54)	< .0001	134	34,853	78,840	43,841,756
Doxycycline	1.48	(1.23–1.78)	0.0001	110	41,549	78,864	43,835,060
Levofloxacin	1.59	(1.44–1.75)	< .0001	429	150,575	78,545	43,726,034
Moxifloxacin	2.84	(2.50–3.22)	< .0001	239	47,007	78,735	43,829,602

a Number of reports of taste and smell disorders due to antibiotics, b: Number of reports of taste and smell disorders other than antibiotics, c: Number of reports of other AEs due to antibiotics, d: Number of reports of other AEs other than antibiotics. The results of the analysis of clindamycin and moxifloxacin indicate those of clindamycin hydrochloride and moxifloxacin hydrochloride, respectively. (see Supplementary Table S1-S2.)

Since it was confirmed that similar results could be obtained when the total number of reports was added up, the maximum reported component of each antibiotic was used as the representative value in this study (see Supplementary Table S2).

**Figure 2:**
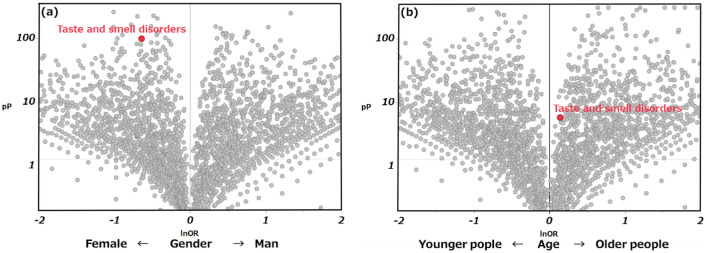
Relationship between antibiotic-induced taste and smell disorders and gender/age. Volcano plots of taste and smell disorders and gender differences/age due to antibiotics. (**a**) It was suggested that this was more likely to occur in women than in men. (**b**) Older people are defined as being over 60 years old. It was suggested that it is more likely to occur in older people over 60 years old than in younger people.

**Figure 3:**
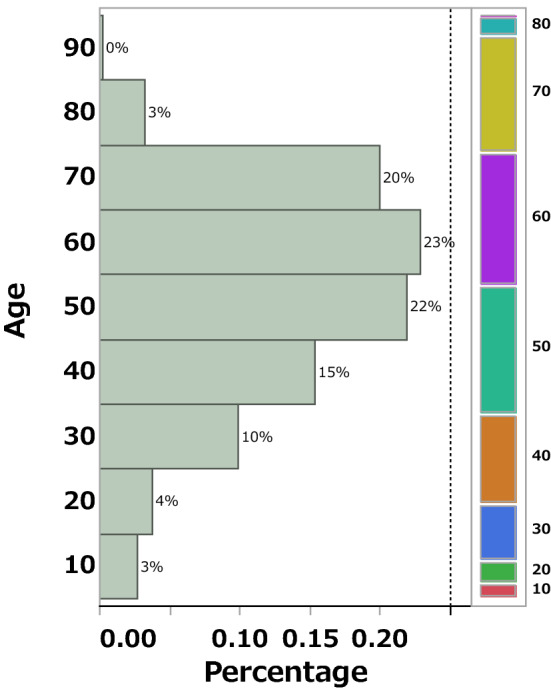
Histogram of age in antibiotic-induced taste and smell disorders. Age was stratified into 10-year groups. The feature of taste and smell disorders peaked in the 60 s (23%), followed by the 50 s (22%), 70 s (20%), and 40 s (15%).

Furthermore, to comprehensively examine the feature of taste and smell disorders by gender, volcano plots were created to show the relationship between ROR and the *P* value for each AEs. The results illustrated that antibiotic-induced taste and smell disorders was more common in women than in men (Fig. 2, Table 2). Next, we created a volcano plot for older and younger individuals’ difference, and comprehensively investigated the tendency of taste and smell disorders with age. The results demonstrated that older individuals, defined as an age of at least 60 years, were more likely to develop taste and smell disorders than younger individuals. When defined at the ages of 65 and 70, no significant results were obtained, and the number of reports was biased. Based on these results, the older group comprised people aged 60 years and older (Fig. 2, Table 2). Comparing the number of reports in each age group, taste and smell disorders was frequent among patients in their 50 s to 70 s (Fig. 3).

**Table 2:** Relationships of antibiotic-induced taste and smell disorders with gender and age.

	%	ROR	95%CI	*P* value	a	b	c	d
Gender (man)	0.5	0.5	(0.49–0.56)	< .0001	1493	3194	949,502	1067421
Older people (≧ 60)	0.5	1.2	(1.09–1.22)	< .0001	2293	2394	915,797	1101126
Older people (≧ 65)	0.4	1	(0.95–1.07)	0.762	1726	2961	738,281	1278642
Older people (≧ 70)	0.3	0.9	(0.88–1.00)	0.057	1216	3471	548,159	1468764

a: Number of cases of taste and smell disorders in older males. b: Number of cases of taste and smell disorders in younger females. c: Number of other AEs in older males. d: Number of cases of other AEs in younger females.

**Figure 4:**
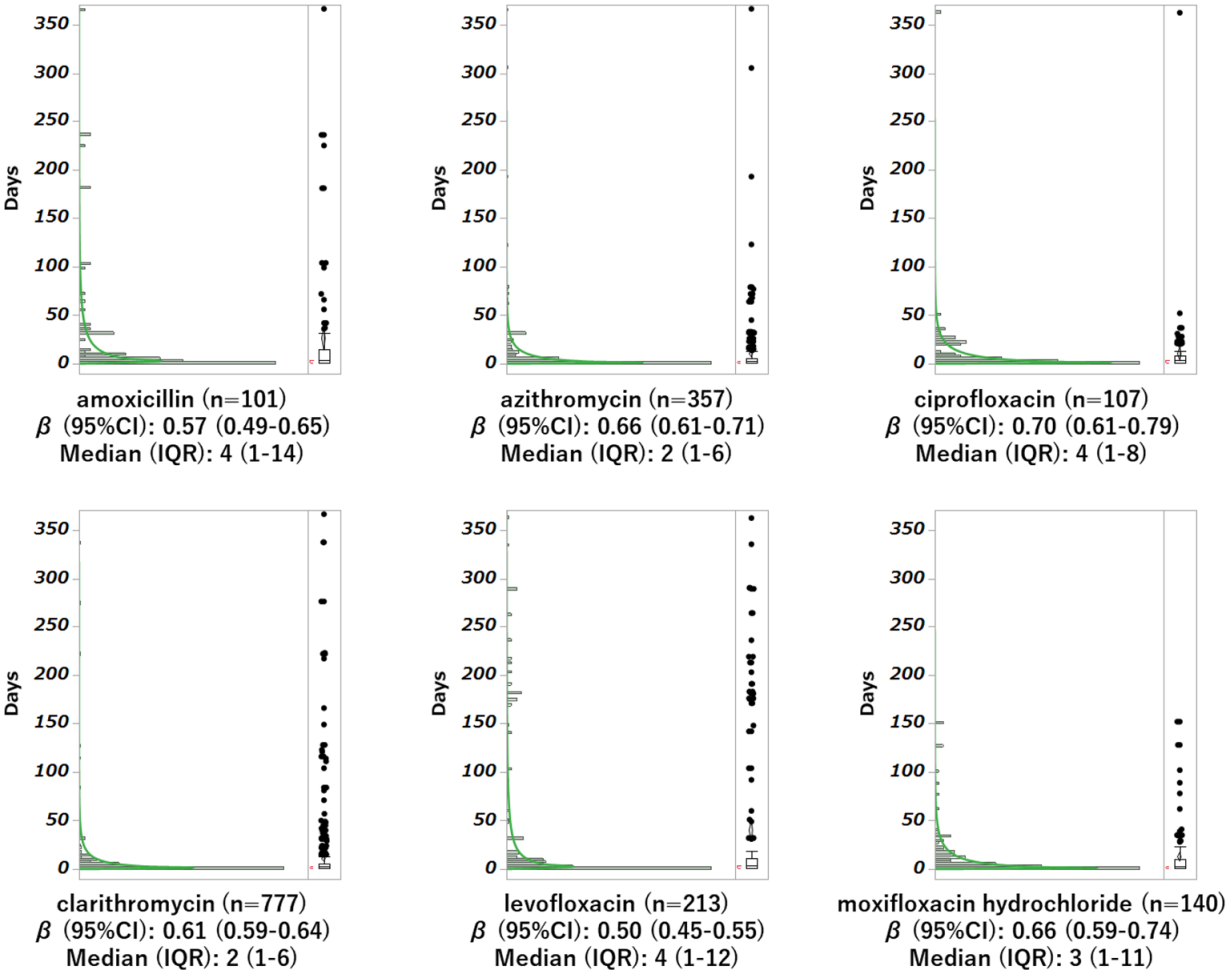
Histogram of antibiotic-induced taste and smell disorders and weibull shape parameters. Adverse event onset time analysis was performed up to 366 days. The median (box center line) and the 25% and 75% quartiles (box ends) are shown to the right of each histogram. The whiskers represent the extreme points ± 1.5 from both ends of the box and represent the largest and smallest values (excluding outliers) of the data within the range. The reliability diamond contains the mean and 95% confidence interval. Parentheses outside the box indicate the shortest range in which 50% of the data are clustered.

Concerning the time to onset of taste and smell disorders, the median duration of antibiotic therapy was 1 week (inter quartile range: 1–6 days). Because the calculated β was less than 1 and the 95% CI did not include 1, the onset of taste and smell disorders was found to decrease over time. (Fig. 4, Table 3).

**Table 3:** β by fitting of weibull distribution.

	Scale parameter		Shape parameter		Median (day)	Interquartile range		n
Drug	α	95%CI	β	95%CI		25%	75%	
Amoxicillin	36.62	(23.0–57.7)	0.42	(0.37–0.48)	5	2	42	117
Azithromycin	7.45	(6.06–9.13)	0.54	(0.50–0.57)	2	1	6	361
Ciprofloxacin	9.07	(6.47–12.6)	0.6	(0.53–0.68)	4	1	8	109
Clarithromycin	10.23	(8.56–12.2)	0.41	(0.40–0.43)	2	1	7	810
Levofloxacin	29.13	(20.8–40.6)	0.41	(0.38–0.45)	5	1	32	229
Moxifloxacin hydrochloride	13.24	(8.97–19.4)	0.45	(0.40–0.50)	4	1	12	145

The calculated β was below 1, and the 95% CI did not include 1. The occurrence of these antibiotic-induced taste and smell disorders was presumed to be an early failure type that is likely to occur early.

AdORs were calculated after controlling for covariates using multiple logistic regression analysis. As a result, AdOR for indicated diseases of hypertension, psychiatric disorders and cancer was greater than 1.0 and above the lower limit of the 95% CI concomitant disease. In addition, the antibiotic AdORs were greater than 1.0 and above the lower limit of the 95% CI for azithromycin, clarithromycin, doxycycline, levofloxacin, and moxifloxacin (Table 4). The *P* value of the estimated model was significant at *P* < 0.0001. The fit of the model was confirmed by the goodness of fit test and the estimated model was appropriate.

**Table 4:** AdORs and *P* values as determined via multiple logistic regression analysis.

	AdOR	95% CI	*P* value
Repoting year	0.99	(0.98–1.00)	0.002
Older people (≧ 60)	1.17	(1.13–1.21)	< .0001
Gender (man)	0.88	(0.85–0.91)	< .0001
(Con) hypertension	1.06	(1.00–1.11)	0.043
(Con) circulatory disease	0.59	(0.54–0.64)	< .0001
(Con) mental disorder	1.09	(1.02–1.16)	0.014
(Con) renal disease	0.35	(0.24–0.47)	< .0001
(Con) cancer	1.49	(1.44–1.55)	< .0001
Azithromycin	2.78	(1.94–3.84)	< .0001
clarithromycin	4.14	(3.24–5.20)	< .0001
Clindamycin hydrochloride	1.44	(0.52–3.13)	0.415
Doxycycline	4.8	(3.00–7.23)	< .0001
Levofloxacin	1.67	(1.19–2.27)	0.002
Moxifloxacin hydrochloride	2.4	(1.47–3.68)	2E − 04

(Con): concomitant diseases.

Spearman's squared rank correlation coefficient (ρ) was less than 0.9 for all combinations. For this reason, no internal correlation was observed among the items used in this analysis, and thus, they were treated as independent factors.

The need to evaluate the possibility of false signals owing to confounding factors in the analysis of adverse drug reaction databases in FAERS has been highlighted^19^. We calculated signal intensity adjusted for confounding factors to account for the possibility of detecting a false signal owing to such confounding factors (Table 5). We concluded that clindamycin was a false signal. A decrease in signal intensity was observed for three antibiotics that did not result in a false signal, confirming that confounding factors may have resulted in an increased signal intensity estimate. Furthermore, these results were like those of AdOR calculated by logistic regression analysis.

**Table 5:**
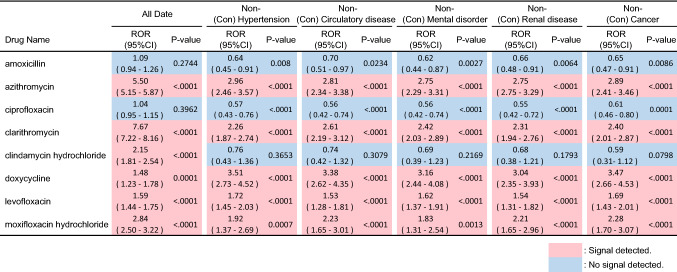
ROR of antibiotics that cause taste and smell disorders adjusted for the effects of concomitant disease. A signal was determined to be present if the lower limit of the 95% CI of the recalculated ROR was greater than 1. If a signal was determined to be undetectable after adjustment for confounding factors, the signal intensity of that drug before adjustment was a false signal due to confounding factors.

## Discussion

The study detected signals for taste and smell disorders for six antibiotics. However, the possibility of a false signal was considered regarding clindamycin. For some of these drugs, taste and smell disorders is not listed as an AE in the package insert in Japan. Fluoroquinolone antibiotics are known to inhibit zinc absorption by forming chelates with zinc. In addition, tetracycline antibiotics are also known to inhibit zinc absorption^20^. In addition, it has been reported that macrolide antibiotics decrease the blood levels of divalent cation elements (calcium and magnesium)^21^. Based on the interactions among these metal cations and antibiotics, it is considered that taste cell turnover^22^, which is generally considered to require approximately 10 days, is delayed, resulting in taste and smell disorders. Additionally, drug-induced olfactory disturbances are thought to be caused by damage to the olfactory nerve itself, which is in the olfactory mucosa and can cause olfactory neuropathy. It has been suggested that gender might affect the onset of drug-induced taste and smell disorders^23^. Some reports in Japan revealed that its incidence is higher in women^6^, in line with our findings. Regarding the spontaneous reporting system, it is more frequently reported in women than in men, which may affect the signal intensity. However, based on the results of this study, caution must be exercised when administering the drug to women.

Generally, drug-induced AEs are more likely to occur in older individuals than in younger individuals. Past research reported that the risk of taste and smell disorders increases with aging^24^. This finding is believed to be attributable to nutritional deficiency caused by decreases in appetite and salivary secretion, resulting in decreased zinc absorption and delayed taste cell turnover. Given that antibiotics can inhibit the absorption of zinc^25^, it is possible that these drugs have direct effects on taste buds in the oral cavity.

Conversely, older individuals are more frequently prescribed drugs that reduce salivation, and they are more frequently prescribed multiple drugs simultaneously^26^. In addition, changes in saliva properties with aging can affect taste and taste performance^27^. In addition, age-related loss of olfactory cells and neurodegenerative diseases, such as Alzheimer's disease, can affect the sense of smell and olfactory performance. The increased risk of the development of antibiotic-induced taste and smell disorders in patients over 60 years of age identified in the present study confirms the results of our previous study^7^. This analysis revealed that patients with a history of hypertension, psychiatric disorders and cancer were more likely to develop drug-induced taste and smell disorders. These factors may be associated with treatment with anticholinergic drugs, ARB/ACE inhibitors, and chemotherapy and other anti-cancer treatments. Taste and smell disorders have also been associated with antibiotics marketed for ophthalmic use (0.5% of the reports of taste and smell disorders for which the route of administration could be ascertained). Some ophthalmic preparations marketed in Japan have taste and smell disorders listed as side effects in the package inserts, and although the frequency of such cases is considered low, caution is considered necessary for ophthalmic preparations as well. The purpose of the analysis of AE onset time was to compare the risk and developmental profile of taste and smell disorders in the clinic using new information^28^. The present study revealed that the onset of antibiotic-induced taste and smell disorders peaked at 1 week and decreased over time. Based on these findings, we recommend that patients taking fluoroquinolone, macrolides, or tetracycline antibiotics be monitored for at least 1 week and then followed for about 2 months. This study had some limitations. SRSs such as FAERS are affected by over-reporting, under-reporting, missing data, the exclusion of healthy individuals, the lack of denominator, and the presence of confounders. Thus, such studies cannot quantify the true risk. For example, ROR examines only the increased risk of AE reporting but not the risk of AE occurrence. Therefore, care must be taken in interpreting the results from the FAERS database. In the case of taste and smell disorders, there is a possibility of under-reporting due to the nature of subjective symptoms. In addition, no analysis of antibiotic dosage was conducted, so it is not clear what the dosage limit is. Moreover, information on the route of administration, concomitant medications, and antibiotic combination therapy were not analyzed in this study. These factors may affect the development of taste and smell disorders and should be considered confounding factors and should be addressed in future studies. This study analyzed taste and smell disorders by stratifying the cases. On the other hand, since patients may contribute to multiple reports in FAERS, furth Alzheimer insights may be gained by analyzing indications and concomitant medications as covariates. Since these analyses may shed light on the effects of disease and concomitant medications on taste and smell disorders, we are considering conducting further analyses.

## Conclusion

This is the first study to evaluate the association between antibiotics and taste and smell disorders using the FAERS database and to analyze the onset of taste and smell disorders induced by individual antibiotics. Finally, accurate signals of antibiotic-related taste and smell disorders were detected for five drugs. In addition, taste and smell disorders is not listed as an AE in the package inserts of some of the identified drugs. The relationship between patient information and antibiotic-induced taste and smell disorders confirms observations from previous studies regarding gender and age differences. The weibull distribution analysis indicated that the identified antibiotics cause an early type of taste and smell disorders. The results of this study stress the need for careful patient monitoring after antibiotics are prescribed. It should also help minimize the risk of the development of antibiotic-induced taste and smell disorders to develop.

## Methods

### Creating a data table

FEARS is a database that uses the large-scale SRS published by the US FDA. We performed this analysis using JAPIC AERS provided by the Japan Pharmaceutical Information Center (JAPIC). Data from November 1997 to March 2017 were included.

JAPIC AERS is data cleaned and provided by JAPIC so that it can be collated and analyzed using the FAERS data published by the U.S. FDA, which operates FAERS. Data are only available for contracted customers^29^.

FEARS consist of seven data tables (DRUG), adverse event information (REAC), case information (DEMO), treatment information (THER), underlying disease information (INDI), outcome information, and reporter information. The DRUG, REAC, DEMO, THER and INDI tables were joined using the ID numbers assigned to each of these tables. In the DRUG table, drugs are classified into four categories, first suspected drug (PS), second suspected drug (SS), concomitant drug, and interaction, according to the degree of involvement in the expected AE. In the JAPIC AERS, data entries in the THER table are treated as missing data if they cannot be converted to the eight-digit date type (YYYYMMDD); hence, in the DEMO table and THER table, 4,486,811 of the 8,597,728 entries (48%) and 2,372,833 (78%) of the 10,781,670 cases were reported in the "YYYYMMDD" format, respectively.

In this study, only reports recorded in PS and SS were used. Antibiotics were extracted from the drugs to be analyzed using systemic antibacterial drugs (J01) based on the WHO Anatomical Therapeutic Chemical Classification System. JAPIC AERS contains JAPIC dictionary files that can be linked to DRUG tables (Medicinal Products—Names of Components, Medicinal Products—ATC Classification [Anatomical Therapeutic Chemistry]). Reports for genders other than male and female have been deleted. Age only used reports recorded in “YR,” unknown data or data for patients beyond the age of 130 years were deleted. We also differentiated age into younger individuals and older individuals based on age 60.

### Definition of taste and smell disorders

Diseases in the REAC and INDI tables are based on medical terms as preferred terms (PTs) in the ICH International Medical Dictionary for Regulatory Activities/Japanese (MedDRA/J). The MedDRA Standard Query (SMQ) is a group of MedDRA terms related to a defined medical condition or area of interest.

In this study, “Taste and smell disorders (SMQ: 20,000,046)” described in MedDRA/J ver. 18.0 were analyzed. The SMQ contained 12 PTs (Table 6). These extracted PTs were designated as "taste and smell disorders."

**Table 6:** Definition of taste and smell disorders.

Taste and Smell disorders (SMQ:20000046)	
PT	
10001480	Ageusia
10002653	Anosmia
10013911	Dysgeusia
10064480	Gustometryabnormal
10019071	Hallucinationgustatory
10019072	Hallucination, olfactory
10069147	Hypergeusia
10020989	Hypogeusia
10050515	Hyposmia
10056388	Olfactorynervedisorder
10062927	Olfactorytestabnormal
10034018	Parosmia

In the extraction by “Taste and smell disorders (SMQ: 2,000,046)”, 12 preferred terms (PTs) were included, including six PTs related to taste and six PTs related to smell.

### Time to AE onset

In the analysis of the time to onset of AEs, only data for patients who reported taste and smell disorders among those treated with antibiotics were extracted. The number of days required to develop taste and smell disorders was calculated from the administration start date of the suspect drug reported in FAERS and the onset date of taste and smell disorders^17,26^. The weibull shape parameter test is used to analyze time-to-onset data, and it can describe the non-constant rate of AE feature. Unknown reports on the date of treatment initiation or the onset of AEs were deleted. In this study, we analyzed more than 100 reported antibiotics and calculated the number of days until AE onset by subtracting the date of drug initiation from the date of AE onset. One was added to the calculated number of days because the start date of the drug was set as day 1. The upper limit of time to AE onset was set to 366 days.

### Multiple logistic regression analysis

AdORs were calculated by the covert adjustment method using a multiple logistic regression equation to correct for the influence of patient background^12,14,15^. Based on a report by Syed et al.^1^, age, gender, and concomitant disease (hypertension, cardiovascular disease, cancer, mental disorder, renal failure) were used as coverts.

Hypertension, cardiovascular disease, cancer, mental disorder, and renal failure comprised the concomitant disease for multiple logistic regression analysis considering the patient background (Table 7). SMQ and Organ Classification were used to extract concomitant disease data. The estimated models were evaluated by the goodness of fit test.

**Table 7:** Definition of cancer history.

(Con) Hypertension		(Con) Circulatory disease		(Con) Cancer	
SMQ		SMQ		SMQ	
20000130	Pulmonary hypertension	20000001	Torsades de pointes/QT prolonged	20000092	Malignant disorder-related state
20000147	Hypertension	20000004	Heart failure	20000094	Tumor marker
		20000047	Myocardial infarction	20000110	Neoplasm of the oropharynx
		20000051	A laboratory study, a sign associated with arrhythmia and symptom	20000194	Malignant tumor
**(Con) Mental disorder**		20000055	Sinus node dysfunction	20000195	Tumor unidentified in detail
SMQ		20000056	Conduction disorders	20000196	Malignant biliary tract neoplasm
20000117	Psychosis and psychopathic disorder	20000057	Supraventricular tachyarrhythmia	20000197	Biliary tract neoplasm unknown in detail
20000142	Hostility/aggressiveness	20000058	Ventricular tachyarrhythmia	20000198	Malignant breast tumour
20000167	Depression (except suicide/self-harm)	20000067	Shock-related circulation or heart state (except torsades de pointes)	20000199	Breast tumor unknown in detail
		20000068	Torsades de pointes, arterial embolus that is in a shock-related state and thrombus	20000200	Malignant ovarian tumor
		20000082	Arterial embolus and thrombus	20000201	Ovarian tumor unidentified in detail
**(Con) Renal disease**		20000083	Of unknown vascular type or mixed embolus and thrombus	20000202	Malignant prostate tumor
SMQ		20000084	Venous embolus and thrombus	20000203	Prostate tumor unidentified in detail
20000003	Acute renal failure	20000150	Cardiomyopathy	20000204	Malignant skin tumor
20000181	Renal vessel disorder	20000162	Nonspecific arrhythmia term	20000205	Skin tumor unidentified in detail
20000213	Chronic kidney disease	20000163	Nonspecific bradyarrhythmia term	20000206	Malignant uterus/salpingioma
		20000164	Nonspecific tachyarrhythmia term	20000207	Uterus/salpingioma unidentified in detail
		20000168	Other ischemic heart disease	20000208	Malignant hepatophyma
				20000209	Hepatophyma unidentified in detail
				20000215	Malignant lymphoma

Concomitant disease examined hypertension, cardiovascular disease, cancer, mental disorder, and renal failure. Each of these combined multiple SMQs and defined them as one concomitant disease. One group was used for hypertension, three groups for mental disorder, three groups for renal failure, 15 groups for cardiovascular disease, and 20 groups for cancer.

(Con): concomitant diseases.

### Calculation of signal intensity adjusting for confounding factors

Reports of concomitant disease that were associated with taste and smell disorders were excluded from the analysis. After removal of concomitant diseases, signal intensity was re-evaluated to assess false positive results. Concomitant diseases were defined as hypertension, cardiovascular disease, cancer, mental disorder, and renal failure. If the signal was determined to be undetectable after adjustment for concomitant diseases, the signal intensity for that drug before adjustment was determined to be a false signal due to confounders.

### Assessment of false positives in signal detection

For the history of the current disease, the target disease was selected by multiple logistic regression analysis. A signal was considered present if the lower limit of the 95% CI of the recalculated ROR was > 1. If a signal was considered undetectable after adjustment for confounding factors, the signal intensity of that drug before adjustment was deemed to be a false signal due to confounding factors.

### Analytical method

**Figure 5:**
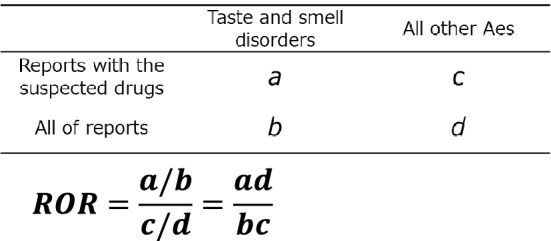
Cross-tabulation and calculation formula of for the reporting odds ratio (ROR) of taste and smell disorders. (**a**) Number of cases of taste and smell disorders attributable to the suspected drug. (**b**) Number of cases of taste and smell disorders attributable to other drugs. (**c**) Number of cases of other AEs attributable to the suspected drug. (**d**) Number of cases of other AEs attributable to other drugs. ROR was calculated using the presented formula.

Based on the two classifications of taste and smell disorders and suspected drugs, a two-by-two frequency table was prepared, ROR and 95% CI (95% CI) were calculated, and Fisher's direct exact test was performed. We compiled a cross-tabulation table based on two classifications: the presence or absence of taste and smell disorders and the presence or absence of the suspected drugs. Therefore, we calculated the *P* values using Fisher’s exact test and RORs (Fig. 5). RORs cannot be calculated if there are cells with 0 in the cross-tabulation table, and the estimation becomes unstable if the frequency is small. To correct for this bias, a correction was made to increase the values in all cells by 0.5 (Haldane-Anscombe correction)^30^.

When the lower limit of the calculated 95% CI of ROR exceeded 1, it was judged that there was a signal^31^. A scatter plot was created (volcano plot) with the logarithm of ROR as the X-axis and the negative logarithm of *P* value calculated using Fisher's exact test as the Y-axis to identify drugs associated with taste and smell disorders^18,32^. In this study, we analyzed antibiotics with more than 100 reports. The time to the onset of taste and smell disorders was calculated as the time from the date of treatment initiation to symptom onset. The scale parameter α of the weibull distribution determines the scale of the distribution function. The shape parameter β of the weibull distribution indicates the hazard without a reference population. When β equals 1, the hazard is estimated to carry a constant risk over time. When β exceeds 1 and the 95% CI of β does not include 1, the hazard risk is considered to increase over time. When β is smaller than 1 and the 95% CI of β does not include 1, the hazard risk is considered to decrease over time^15,17,18,20,28^.

**Figure 6:**
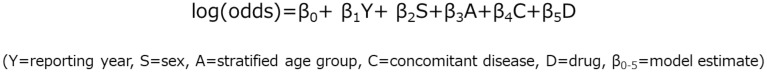
Multiple logistic regression equation for calculating the adjusted odds ratio.

Multiple logistic regression analysis was performed to examine the effects of the analyzed drugs and patient background. AdORs were calculated to control for coverts using a multiple logistic regression equation. Patients were stratified by age into younger (< 60 years) and older populations (60–130 years)^7^. Gender, age, concomitant disease, and drug use were coded using this stratification (Fig. 6).

The analyzed drugs were determined to be significantly associated with taste and smell disorders based on the ROR signal, and the number of reports was at least 100.

The internal correlation was estimated using the pairwise method. If the square of ρ exceeded 0.9, then we concluded that an internal correlation existed. In the absence of an internal correlation, we treated these items as independent factors^12^. To detect false signals, we recalculated the signal intensities after removing suspected confounders related to concomitant disease for the antibiotics included in Table 1. Signal intensities for drugs eliciting taste and olfactory disturbances were calculated with and without the removal of the current history of suspected confounding factors. All data were analyzed using JMP Pro 14.3.0 (SAS, Cary, NC).

## Supplementary Information

Supplementary Information.
